# Development and psychometric assessment of Health Action Process Approach (HAPA) in terms of smoking cessation among Chinese smokers

**DOI:** 10.1038/s41598-024-54404-2

**Published:** 2024-02-19

**Authors:** Hao Lin, Haoxiang Lin, Lanchao Zhang, Chengqian Zhang, Xiaochen Yang, Wangnan Cao, Chun Chang

**Affiliations:** 1 Department of Social Medicine and Health Education, School of Public Health, Peking University Health Science Center, Peking University, Beijing, China; 2 Institute for Global Health and Development, Peking University, Beijing, China

**Keywords:** HAPA, Smoking cessation, Exploratory factor analysis, Validity, Lifestyle modification, Preventive medicine

## Abstract

The Health Action Process Approach (HAPA) is a two-stage (pre-intentional and post-intentional) behavioral change model that distinguishes between motivation and volition in behavior change process. This study aims to develop HAPA-based assessments for smoking cessation among current smokers. The HAPA-based measures were developed and the draft measures included nine constructs, namely, risk perception in smoking-induced cancer, risk perception in smoking-induced systemic disease, positive outcome expectancy, negative outcome expectancy, self-efficacy in quitting smoking, self-efficacy in maintaining, self-efficacy in re-initiating, quitting planning and coping planning in smoking cessation, with a total of 26 items. A cross-sectional survey was conducted in China in 2022. Principal Component Analysis was used for Exploratory Factor Analysis (EFA). Cronbach’s α coefficient was calculated to evaluate the internal consistency. Variables such as severity of smoking addiction were selected to evaluate the correlation between the HAPA scale and these variables. Of the 928 participants, 76.4% (709/928) were male and the median age was 35 years. Five factors were extracted by EFA. The factor loadings of each item were all greater than 0.60, and the cumulative variance contribution rate was 90.15%. The Cronbach’s α coefficient of each HAPA-based subscales was 0.929–0.986. The HAPA-based measurements are comprehensive, reliable and valid in the assessment of smokers’ smoking cessation cognition, which can be used to guide the design and implementation of intervention and the development of theory.

## Introduction

Tobacco smoking is a major public health problem. The World Health Organization (WHO) reports that three million people die prematurely each year from chronic diseases caused by smoking^1,2^. People who quit smoking can significantly reduce the risk of developing and dying from tobacco-related diseases^3^. Most of the smokers are aware of the harm of cigarettes, and more than half make a quit attempt every year^4^. However only less than 10% of those who attempt could remain abstinent for at least 6 months^5,6^. In addition, approximately 10% of quitters relapse annually^7^.

Theory-based interventions were more likely to succeed in changing behaviors comparing to those without theoretical background^8^. A range of health behavior and behavior change theories are utilized to explain the mechanisms of human behavior change and to promote behavior change^9^. Previous theories that have been tested in the domain of smoking cession include the Theory of Planned Behavior (TPB), Protection Motivation Theory (PMT), and Health Belief Model (HBM). Most of the studies reported significant effects of theory-based interventions on smoking related outcomes, including smoking behaviors, intentions and attitudes^10–14^. However, given that smoking cessation is a dynamic daily process, most of traditional single-stage models of behavior change fail to clearly account for the continuously frequent fluctuations, stages of change, self-regulatory processes, or the causal order among the predictors^15^. TPB has the assumption that behavioral intention is a predictive factor for a subsequent behavior, but it fails in illustrating how to transform the behavioral intention into an actual behavior^10^. This intention-behavior gap is particularly common in the context of smoking cessation, and Health Action Process Approach (HAPA) is a more useful framework in bridging this gap (compared to TPB). Compared to PMT, HAPA divided smokers into those having smoking cessation intentions and those without, and this distinction can help intervention design more tailored and programs more easily to be succeed.

HAPA postulates a heuristic assembly of social-cognitive variables and makes a distinction between pre-intentional and post-intentional processes, which makes it particularly applicable in the context of smoking cessation interventions. More than two-thirds of smokers reported that their thoughts about quitting changed daily, and such fluctuations often predict cessation lapse in smokers undergoing a quit attempt^16^. Another strength of HAPA is that its post-intentional phase specifies how intentions can be converted into behaviors by addressing the intention-behavior gap^17^, and this advantage in operationalization helps to provide targeted information in smoking cessation interventions.

As one of the stage-based behavioral theories, the HAPA is a social-cognitive model specifying motivational and volitional determinants of health behavior. Behavioral intention is a key element to initiate behavior in HAPA. Taking behavioral intention formation as the demarcation point, the process of behavioral change can be divided into the pre-intentional stage (also known as the motivational stage) and the post-intentional stage (volitional stage)^18^. Self-efficacy is crucial in all stages of action, while risk perception and outcome expectancy mainly play a role in the motivational stage^19^. After making individual decisions, the perception and coping of obstacles become important factors to promote the maintenance of behavior^17^.

HAPA has been widely tested in different types of health behaviors, including healthy diet, physical exercise and flossing^20–22^, but HAPA is rarely investigated in smoking cession^23^. The study by Williams et al. generally supports the HAPA prediction that increased risk perceptions would foster the decision to quit smoking^24^. According to the research of Scholz and colleagues based on HAPA theoretical basis, change in action planning and especially action control was of great importance for smoking behavior change^25^.

We believe that HAPA has its potential applicability in smoking cessation for several reasons. First, some of the HAPA-based constructs, such as self-efficacy, outcome expectancy, planning, and action control, have been identified as key factors in quitting smoking^23^. For example, positive outcome expectancies of quitting smoking and the belief in one’s capability to quit smoking were associated with one’s motivation to reduce the number of cigarettes smoked^26^. Second, when it comes to identifying individuals at different stages along the health behavior change process, the HAPA proposes a subdivision of the volitional phase. It emphasizes the importance of not only motivating individuals to quit smoking but also providing them with the necessary skills and strategies to maintain long-term abstinence. In the context of smoking cessation, some smokers who have quitting attempts or quitting intentions are unable to sustain abstinent or transfer quitting intention to quitting behavior; such relapse and intention-behavior gap make HAPA particularly relevant. Third, the stage-specific approach of HAPA increases the relevance and effectiveness of interventions. It provides tailored interventions that are specific to each stage, taking into account the unique challenges and needs of individuals at different points in their process of quitting. All HAPA-based constructs are highly modifiable, which is one of the key concerns in designing and implementing intervention programs.

As a part of a randomized controlled trial to test the effectiveness of a HAPA-based intervention in smoking cession, the aim of the current study was to develop and validate HAPA-based measures related to smoking cessation among current smokers in China, for a longer-term goal of providing validated measures in assessing the effect of the HAPA based intervention.

## Method

### Study participants and data collection

Data were collected through an anonymous cross-sectional electronic questionnaire survey in July 2022. Adult current smokers (who were defined as smoke at least one cigarette a week) were considered eligible for participation. Participants were recruited by advertisements and referrals. The majority of the participants were from Jiangsu and Shandong provinces in China. Participants took approximately 8.6 ± 6.4 (Mean ± SD) minutes to complete the questionnaire. The completeness and logics of the questionnaire was checked upon submission. Participants were offered CNY10 (= USD1.4) as a compensation for their time spent on this study.

### Measurements

The questionnaire was consisted of four sections, including socio-demographics, current smoking status and past quitting attempts, HAPA-based measures related to smoking cessation, and psychosocial status.

#### Current smoking status and past quitting attempts

Current smoking status were measured by variables including severity of smoking addiction and smoking frequency. The Fagerstrom Test for Nicotine Dependence (FTND) was used to measure nicotine dependence and severity of smoking addiction^27^. In addition, items such as the number of attempts to quit smoking, duration of quitting smoking, and quitting method were measured to evaluate the past quitting attempts of participants.

#### HAPA-based measures related to smoking cessation

We conducted literature review and expert consultation in drafting the HAPA-based items/constructs. For the literature review, we emphasized on smoking-based cognitions and HAPA-based measures on other health behaviors and constructed an item pool. For the expert consultation, a research panel with multidisciplinary backgrounds reviewed key literatures and discussed all potential items (and constructs) in several meetings. For smoking cessation intention, the willingness to quit smoking in the next 6 months was measured (Likert scale, 5: very likely, 1: very unlikely). Under the mainly reference of the scale developed by Joveini, et al.^28^, nine HAPA-based constructs (with mostly three items for each construct) were drafted, including:

1. Risk perception in smoking-induced cancer, one example item was “what are my chances of getting lung cancer due to smoking?”;
2. Risk perception in smoking-induced systemic disease, one example item was “what are my chances of having cardiovascular diseases due to smoking?”;
3. Positive outcome expectancy if quit smoking, one example item was “my physical condition will be improved”;
4. Negative outcome expectancy if quit smoking, one example item was “stop smoking prevents me from socialization”;
5. Self-efficacy in quitting smoking, one example item was “I can start quitting smoking, even if I feel tense and nervous”;
6. Self-efficacy in maintaining smoking cessation, one example item was “I can continue not to smoke, even if I have severe withdrawal symptoms”;
7. Self-efficacy in re-initiating smoking cessation after relapse, one example item was “I can quit again, even if I have postponed my cessation program several times”;
8. Quit smoking planning, one example item was “I have a precise plan concerning the time of initiating smoking cessation”;
9. Coping planning in maintaining smoking cessation, one example item was “I have a clear plan on how to avoid smoking places”.

The item responses were rated on 5-point Likert- scales from 1 (strongly disagree) to 5 (strongly agree) (see Table 1 for full details).

**Table 1:** Descriptive statistics for the items in the Health Action Process Approach constructs (n = 928).

Construct/question		Score range	Mean	SD	Floor effect (%)	Ceiling effect (%)	Skewness	Kurtosis
Risk perception in smoking-induced cancer								
A1	What are my chances of getting lung cancer due to smoking?	1–5	4.41	0.94	2.91	63.36	− 1.87	3.37
A2	What are my chances of getting month cancer due to smoking?	1–5	4.31	1.01	2.91	59.38	− 1.52	1.73
A3	What are my chances of getting bladder cancer due to smoking?	1–5	4.19	1.09	3.23	56.14	− 1.20	0.57
Risk perception in smoking-induced systemic disease								
B1	What are my chances of having cardiovascular diseases due to smoking?	1–5	4.33	0.98	2.80	59.48	− 1.59	2.13
B2	What are my chances of having respiratory diseases due to smoking?	1–5	4.40	0.94	2.69	61.96	− 1.79	3.03
B3	What are my chances of having reproductive diseases due to smoking?	1–5	4.19	1.10	3.34	56.57	− 1.22	0.59
Positive outcome expectancy if quit smoking								
C1	My physical condition will be improved	1–5	4.04	1.22	6.90	50.43	− 1.16	0.37
C2	My smoking-related expenses will be reduced	1–5	4.05	1.21	6.68	50.22	− 1.18	0.43
C3	My family and friends will be happy	1–5	4.07	1.23	7.33	52.80	− 1.24	0.52
Negative outcome expectancy if quit smoking								
D1	Stop smoking prevents me from socialization	1–5	2.86	1.30	19.94	14.98	0.12	− 0.97
D2	Stop smoking deprives me of an opportunity for enjoyment	1–5	2.83	1.33	21.98	14.66	0.12	− 1.04
D3	Stop smoking makes me disable to deal with stress	1–5	2.91	1.31	19.72	15.19	0.05	− 1.02
Self-efficacy in quitting smoking								
E1	I can start quitting smoking, even if I feel tense and nervous	1–5	3.73	1.14	4.42	33.08	− 0.51	− 0.51
E2	I can start quitting smoking, even if I have a strong temptation to smoke	1–5	3.73	1.13	4.09	33.62	− 0.48	− 0.58
E3	I can start quitting smoking, even if my significant others do not support me to quit	1–5	3.72	1.14	4.63	33.19	− 0.52	− 0.51
Self-efficacy in maintaining smoking cessation								
F1	I can continue not to smoke, even if I have severe withdrawal symptoms	1–5	3.74	1.13	4.20	32.97	− 0.52	− 0.49
F2	I can continue not to smoke, even if I feel tense or restless or tired	1–5	3.73	1.13	4.20	32.87	− 0.51	− 0.50
F3	I can continue not to smoke, even if my friends offer me a cigarette	1–5	3.79	1.08	3.02	33.94	− 0.49	− 0.49
Self-efficacy in re-initiating smoking cessation after relapse								
G1	I can quit again, even if I have postponed my cessation program several times	1–5	3.80	1.07	3.02	33.51	− 0.51	− 0.42
G2	I can quit again, even if I am not able to refrain from smoking sometimes	1–5	3.80	1.07	3.13	33.30	− 0.52	− 0.39
G3	I can quit again, even if I returned to smoking and abandoned the cessation program	1–5	3.80	1.09	3.02	34.27	− 0.52	− 0.48
Quit smoking planning								
H1	I have a precise plan concerning the time of initiating smoking cessation	1–5	3.72	1.14	3.99	33.41	− 0.46	− 0.64
H2	I have a precise plan concerning the process of initiating smoking cessation	1–5	3.71	1.14	3.77	33.19	− 0.44	− 0.67
Coping planning in maintaining smoking cessation								
I1	I have a clear plan on how to avoid smoking places	1–5	3.74	1.11	3.34	33.08	− 0.46	− 0.61
I2	I have a clear plan on how to cope with temptations to smoke	1–5	3.75	1.11	3.45	33.30	− 0.48	− 0.56
I3	I have a clear plan on how to overcome the situation which makes me more likely to start smoking again	1–5	3.74	1.11	3.34	33.08	− 0.44	− 0.62

SD, standard deviation.

#### External variables for validation

Based on previous literature, the social relationships and mental health symptoms of participants may be directly or indirectly associated with cigarette dependence, perceived barriers for cessation, and smoking reduction^29–31^. Therefore, these external variables, such as social support, depression, and anxiety, were measured to assess the participants’ psychosocial status and determine the correlation between the HAPA scale and these validated variables. This was done to verify whether the scale could accurately capture agreement with these relevant variables and further evaluate the usefulness of the HAPA scale. Social support was divided into emotional support and instrumental support^32^, and we measured these two different types of support offered by spouse (if any), family, friends and colleagues, respectively. Depression was measured by CES-D-10 (Center for Epidemiologic Studies Depression Scale)^33^ and anxiety was measured by GAD-7 (Generalized Anxiety Disorder)^34^.

### Content validity assessment

Content validity was qualitatively evaluated by a research panel with multidisciplinary backgrounds (epidemiology, behavioral health, and psychology). A careful review including evaluation of the wording and placement of each item within the scale was performed. The corrective views were applied point by point to the questionnaire, and the items which were considered ambiguous were reformulated according to the suggested improvements.

### Face validity assessment

The research team conducted qualitative face validity assessments in the form of face-to-face interviews with residents of nearby communities. After being provided with the necessary explanations about the research, participants were asked to comment on the comprehensibility and the clarity of each questionnaire item. Necessary corrections were made in response to these comments^35^.

### Statistical analysis

Descriptive statistics (means and standard deviation) were presented for all items. The floor effect (at the very low end of the scale) and ceiling effect (at the very high end of the scale) were examined^36^; an item was considered non-responsive if its floor or ceiling effects exceeded 70%. The factor structure was assessed by using the Exploratory Factor Analysis (EFA)^37^. The Kaiser–Meyer–Olkin (KMO) Measure of Sampling Adequacy and Bartlett’s Test of Sphericity were applied to measure sampling adequacy and to examine the appropriateness of the factor analysis. We used the principal component method^38^ for extraction with Oblimin with Kaiser Normalization for rotation (Delta = 0). The criterion for retaining a factor was that it had an eigenvalue higher than 1.0^39^. Only items with factor loadings higher than 0.4 were retained^40^. Those items with factor loadings higher than 0.4 on two or more factors were considered as double loading or overloading; further adjustments (i.e., deleting these items one by one) were conducted^41^. The validity of content and form was qualitatively measured. In addition, stratified analyses of the different genders were conducted based on factor analysis.

Spearman correlation coefficients were computed to examine item-subscale correlations and item-other-subscale correlations considering the non-normality of the data. The item-subscale correlations should be higher than item-other-subscale correlations, indicating that a specific item was properly classified into the current subscale, not the other subscales. The Corrected Item-Total Correlation (CITC) was calculated to evaluate the relationship between individual items and the overall score of the scale. The internal consistency was measured by Cronbach’s alpha coefficients. Spearman correlation coefficients between the constructs of HAPA and six external variables were calculated to determine the correlation between the HAPA scale and these variables^42^. All statistical analyses were performed by using IBM SPSS Statistics (version 27) and SAS (University Edition). The statistical significance level was *p* < 0.05.

### Ethical aspects

The study was approved by the ethics commission of Peking University Health Science Centre (Ethical approval number: IRB00001052-18055). All participants signed an informed consent form before having access to answer the study questions, and all participants were informed about the objectives of the study, guarantee of anonymity and nonuse of the data for other purposes. All research procedures were performed in accordance with the Declaration of Helsinki and other relevant guidelines and institutional regulations applied for studies involving human participants.

## Results

### Descriptive statistics

In this study, a total of 931 current smokers approached the survey, of which 3 completed the survey but the questionnaires were deemed invalid for the consideration of the sample representativeness. Of the remaining 928 participants, around half (53.6%) aged more than 35 years (age range: 18–89 years old); three-quarter (76.4%) were men; the majority (79.0%) attended college or above. Three-quarter (77.5%) of the participants were married and half (56.6%) of them self-reported their monthly income was more than CNY6000 (= USD 840). Two thirds (69.5%) of the participants reported no chronic conditions.

The 26 items of the HAPA-based scale were rated on 5-point Likert-scales from 1 (most unlikely) to 5 (most likely). The highest mean score of the items was 4.41 (SD = 0.94), and the lowest was 2.83 (SD = 1.33). The percentages with floor effect and ceiling effect ranged from 2.7 to 22.0% and 14.66 to 63.4%, respectively (Table 1).

### Factor structures

#### Exploratory factor analysis

Five factors with eigenvalues exceed 1.0 were yielded. The KMO (0.953) and Bartlett’s test (*χ*^2^ = 40363.948, *df* = 325, *p* < 0.001) indicated good performance of the Exploratory Factor Analyses (EFA). The HAPA structures were extracted into factors as follows: (1) Self-efficacy (in quitting smoking, maintaining smoking cessation and re-initiating smoking cessation after relapse) in factor 1; (2) risk perception (in smoking-induced cancer and smoking-induced systemic disease) in factor 2; (3) negative outcome expectancy when quit smoking in factor 3; (4) positive outcome expectancy when quit smoking in factor 4 and 5) planning (quit smoking planning and coping planning in maintaining smoking cessation) in factor 5. The eigenvalues of the factors were 14.62, 3.31, 2.69, 1.82, and 1.01, respectively explaining 56.24%, 12.74%, 10.33%, 6.98%, 3.86% of the total variance (90.15%). The Cronbach’s alpha corresponding to the five factors were 0.983, 0.972, 0.929, 0.958 and 0.986, respectively (Table 2). Stratified analyses based on different genders show that factor analysis results in male smokers were almost consistent with the general population, that is, a total of 5 factors were extracted. The eigenvalues of the factors were 14.48, 3.52, 2.62, 1.67, and 0.90, respectively explaining 55.70%, 13.52%, 10.07%, 6.41%, 3.45% of the total variance (89.16%) (Supplementary Table 1). In female smokers, although also five factors were extracted, there was cross factor loading between the constructs of self-efficacy and planning (Supplementary Table 2).

**Table 2:** Exploratory factor analysis for the Health Action Process Approach.

Construct		Factor 1	Factor 2	Factor 3	Factor 4	Factor 5
Risk perception in smoking-induced cancer						
A1	What are my chances of getting lung cancer due to smoking?	0.02	**0**.**94**	− 0.01	− 0.04	− 0.05
A2	What are my chances of getting month cancer due to smoking?	− 0.01	**0**.**96**	− 0.01	0.00	− 0.01
A3	What are my chances of getting bladder cancer due to smoking?	− 0.07	**0**.**92**	0.02	0.03	0.09
Risk perception in smoking-induced systemic disease						
B1	What are my chances of having cardiovascular diseases due to smoking?	0.04	**0**.**96**	0.00	0.00	− 0.04
B2	What are my chances of having respiratory diseases due to smoking?	0.04	**0**.**94**	− 0.01	− 0.04	− 0.07
B3	What are my chances of having reproductive diseases due to smoking?	− 0.01	**0**.**89**	0.01	0.04	0.08
Positive outcome expectancy if quit smoking						
C1	My physical condition will be improved	− 0.02	0.02	0.00	**0**.**95**	0.01
C2	My smoking-related expenses will be reduced	− 0.01	− 0.01	0.00	**0**.**97**	0.00
C3	My family and friends will be happy	0.01	− 0.02	0.01	**0**.**96**	− 0.02
Negative outcome expectancy if quit smoking						
D1	Stop smoking prevents me from socialization	0.01	− 0.03	**0**.**92**	0.01	0.07
D2	Stop smoking deprives me of an opportunity for enjoyment	0.02	− 0.02	**0**.**95**	0.01	− 0.01
D3	Stop smoking makes me disable to deal with stress	− 0.02	0.05	**0**.**93**	− 0.02	− 0.06
Self-efficacy in quitting smoking						
E1	I can start quitting smoking, even if I feel tense and nervous	**0**.**97**	0.01	0.03	0.00	− 0.04
E2	I can start quitting smoking, even if I have a strong temptation to smoke	**0**.**97**	0.01	0.00	− 0.01	− 0.03
E3	I can start quitting smoking, even if my significant others do not support me to quit smoking	**0**.**98**	0.00	− 0.01	0.00	− 0.03
Self-efficacy in maintaining smoking cessation						
F1	I can continue not to smoke, even if I have severe withdrawal symptoms	**0**.**99**	0.01	0.01	− 0.02	− 0.04
F2	I can continue not to smoke, even if I feel tense or restless or tired	**0**.**98**	0.00	0.01	0.01	− 0.02
F3	I can continue not to smoke, even if my friends offer me a cigarette	**0**.**57**	0.04	− 0.01	− 0.06	0.34
Self-efficacy in re-initiating smoking cessation after relapse						
G1	I can quit again, even if I have postponed my cessation program several times	**0**.**61**	0.08	− 0.02	− 0.03	0.32
G2	I can quit again, even if I am not able to refrain from smoking sometimes	**0**.**62**	0.07	− 0.03	− 0.04	0.31
G3	I can quit again, even if I returned to smoking and abandoned the cessation program	**0**.**60**	0.06	− 0.03	− 0.05	0.33
Quit smoking planning						
H1	I have a precise plan concerning the time of initiating smoking cessation	0.02	0.02	0.00	− 0.01	**0**.**93**
H2	I have a precise plan concerning the process of initiating smoking cessation	0.01	0.01	0.00	0.00	**0**.**96**
Coping planning in maintaining smoking cessation						
I1	I have a clear plan on how to avoid smoking places	0.02	0.02	0.00	− 0.02	**0**.**93**
I2	I have a clear plan on how to cope with temptations to smoke	0.03	− 0.01	0.00	− 0.01	**0**.**94**
I3	I have a clear plan on how to overcome the situation which makes me more likely to start smoking again	0.02	0.04	0.01	− 0.03	**0**.**92**
Cronbach’s alpha		0.983	0.972	0.929	0.958	0.986
Initial eigenvalues		14.62	3.31	2.69	1.82	1.01
Cumulative % of variance explained		56.24	68.97	79.30	86.28	90.15

Extraction Method: Principal Component Analysis. Rotation Method: Oblimin with Kaiser Normalization. Rotation converged in 8 iterations.

Bolded values indicate factor loadings > 0.6, reflecting significant factor indicators.

#### Content validity and face validity assessment

To evaluate the content validity of the scale, we invited five experts to review the items and provide feedback on their relevance, clarity, and comprehensiveness. Based on the experts’ feedback, we reworded some items to make them clearer and more concise. To evaluate the face validity, we conducted a pilot test with 30 participants who completed the questionnaire and provided feedback on their understanding, interest, and difficulty of the items. Based on the participants’ feedback, we simplified the item that was too complex and technical. We changed “I have a clear action plan on how to create a smoke-free work or living environment” to “I have a clear plan on how to overcome the situation which makes me more likely to start smoking again”.

#### Item analysis and internal consistency

Cronbach’s alpha for each subscale was between 0.929 and 0.985. About the item analysis of the Health Action Process Approach, all item-subscale correlation coefficients ranged from 0.897 to 0.974 and all *p* values were less than 0.001. All item-subscale correlation coefficients were higher than those correlation coefficients between the same item and the other subscales (Table 3). Most of the items have CITC between 0.540 and 0.865 (Supplementary Table 3).

**Table 3:** Item analysis of the Health Action Process Approach.

Subscale	Item number	Subscale Cronbach’s alpha if item is deleted	Item-subscale correlation	Item-other-subscale correlation
Risk perception in smoking	A1	0.942	0.939***	0.027–0.499***
	A2		0.953***	0.019–0.502***
	A3		0.924***	0.042–0.495***
	B1	0.940	0.958***	0.027–0.522***
	B2		0.939***	0.030–0.506***
	B3		0.917***	0.029–0.521***
Positive outcome expectancy if quit smoking	C1	0.958	0.957***	0.123–0.441***
	C2		0.967***	0.118–0.425***
	C3		0.956***	0.127–0.413***
Negative outcome expectancy if quit smoking	D1	0.929	0.921***	0.037–0.126***
	D2		0.946***	0.005–0.101***
	D3		0.940***	− 0.006–0.131***
Self-efficacy in quitting, maintaining and re-initiating smoking cessation	E1	0.972	0.925***	0.061–0.746***
	E2		0.944***	0.037–0.767***
	E3		0.940***	0.030–0.758***
	F1	0.948	0.954***	0.045–0.764***
	F2		0.950***	0.048–0.766***
	F3		0.908***	0.020–0.803***
	G1	0.985	0.942***	0.005–0.832***
	G2		0.942***	− 0.001–0.829***
	G3		0.937***	0.001–0.829***
Planning in quit smoking and maintaining smoking cessation	H1	0.971	0.967***	− 0.016–0.815***
	H2		0.977***	− 0.018–0.815***
	I1	0.979	0.976***	− 0.017–0.820***
	I2		0.974***	− 0.013–0.818***
	I3		0.968***	− 0.009–0.814***

*p < 0.05, **p < 0.01, ***p < 0.001.

Item-subscale correlation: Spearman correlation coefficient between each item and its corresponding subscale.

Item-other-subscale correlation: Spearman correlation coefficient between each item and the other subscale.

#### External correlations

Except for the construct D (negative outcome expectancy) which correlated negatively and significantly with the scores of two external variables, smoking cessation intention and social support (Spearman *rs* = − 0.181 to − 0.127, all *p* < 0.001), the scores of other constructs correlated positively and significantly with the aforementioned two scores (Spearman *rs* = 0.180 to 0.377, all *p* < 0.001). Regarding the variables of severity of smoking addiction, CES-D-10 and GAD-7, the scores of constructs other than construct D correlated negatively and significantly with those scores (Spearman *rs* = − 0.324 to − 0.038, all *p* < 0.05), while the scores of construct D correlated positively and significantly with the scores (Spearman *rs* = 0.068 to 0.212, all *p* < 0.05). All constructs correlated negatively and with the variable of number of attempts to quit smoking (Spearman rs = − 0.270 to − 0.197, except for construct C, *p* < 0.001) while construct D correlated positively and significantly with the variable (Spearman *rs* = 0.162, *p* < 0.01) (Table 4).

**Table 4:** Spearman correlation among the Health Action Process Approach and other related variables.

Health Action Process Approach constructs	Severity of smoking addiction	Number of attempts to quit smoking	Willingness to quit smoking	Social support			CES-D-10	GAD-7
				Total support	Total emotional support	Total instrumental support		
Construct A: Risk perception in smoking-induced cancer	− 0.178***	− 0.197***	0.257***	0.231***	0.243***	0.209***	− 0.283***	− 0.257***
Construct B: Risk perception in smoking-induced systemic disease	− 0.180***	− 0.226***	0.259***	0.242***	0.259***	0.217***	− 0.296***	− 0.274***
Construct C: Positive outcome expectancy if quit smoking	− 0.069*	− 0.073	0.180***	0.238***	0.254***	0.212***	− 0.271***	− 0.223***
Construct D: Negative outcome expectancy if quit smoking	0.212***	0.162**	− 0.181***	− 0.144***	− 0.157***	− 0.127***	0.068*	0.110***
Construct E: Self-efficacy in quitting smoking	− 0.269***	− 0.207***	0.334***	0.284***	0.276***	0.277***	− 0.038***	− 0.278***
Construct F: Self-efficacy in maintaining smoking cessation	− 0.268***	− 0.251***	0.329***	0.285***	0.277***	0.280***	− 0.322***	− 0.275***
Construct G: Self-efficacy in re-initiating smoking cessation after relapse	− 0.269***	− 0.266***	0.370***	0.305***	0.306***	0.289***	− 0.324***	− 0.284***
Construct H: Quit smoking planning	− 0.318***	− 0.270***	0.377***	0.329***	0.317***	0.326***	− 0.316***	− 0.284***
Construct I: Coping planning in maintaining smoking cessation	− 0.309***	− 0.250***	0.367***	0.332***	0.319***	0.327***	− 0.320***	− 0.288***

*p < 0.05, **p < 0.01, ***p < 0.001.

CES-D-10, Center for Epidemiological Survey, Depression Scale; GAD-7, Generalized Anxiety Disorder.

## Discussion

In this study, we developed and validated the HAPA-based assessments for smoking cessation in Chinese smokers. The present measure is a comprehensive tool including all HAPA constructs, including those at pre-intentional phase and post-intentional phase. Risk perception was divided into risk perception in smoking-induced caners and smoking-induced systemic diseases, which will help or enrich the current focus on smoking-induced diseases such as lung cancer, month, and bladder cancer, or cardiovascular, respiratory, and reproductive diseases^43–45^. Outcome expectancy (OE) was conceptualized and divided into separate positive and negative dimensions because both of which have a substantially impact to forming an intention and it will make the measurement more comprehensive^46,47^. The positive OE and negative OE cannot be offset^48–50^.

This HAPA-based scale validation study was conducted in a broad population of smokers, and the scale has been shown to be generalizable to the smokers with the current validation, especially the relatively large number of male smokers in the Chinese cultural context. However, the scale needs to be promoted with caution in female smokers, possibly due to the limited sample size of women in this general sample. On the other hand, because female smokers may face more challenges and stress in their social roles, there are differences between female and male smokers in terms of social psychology, economic status and income, etc.^51^. Future studies should consider further development of smoking cessation assessment scales for subpopulations such as female occupational smokers.

One of the advantages of using HAPA to inform future intervention is that the post-intentional phase specifies how to transform intention to behavior by addressing the intention-behavior gap. In the present study, self-efficacy was measured at three different stages in terms of smoking cession: initiating, maintaining, and re-initiating smoking cessation^15,52,53^. Planning was measured at two different stages in terms of initiating and maintaining smoking cessation^54^. We believe separate and tailored measures could be used to put more effective and targeted interventions into action.

Some HAPA based constructs were similar or overlap with part of the constructs in other existing health theories, such as self-efficacy and risk perception; and we found our measures were comparable to them. For example, Pribadi et al. showed that the intention of stop smoking behavior among smokers has a significant correlation with the perceived factors of the HBM construct, which includes perceived susceptibility, perceived severity, perceived benefits, perceived barriers, and perceived self-efficacy^55^. Thrul and his colleagues’ research founded the predictive validity of the coping appraisal construct self-efficacy, one part of the PMT, namely for in predicting smoking-related behavioral intention and smoking behavior^56^.

The present study reported correlations between HAPA-based constructs with some psychosocial variables such as social support and levels of depression and anxiety, which was consistent with the existing literature. For example, Qian et al. showed that the emotional support provided by the community could positively affect achieving smoking cessation goals^57^. In addition, Weinberger et al. found that persons with depression are less likely to quit smoking, and are more likely to relapse^58^.

There were a few limitations for this study that restricted the interpretations of the results: first, given the limited sample size, we did not conduct the Confirmatory Factor Analysis to verify this measure. However, the EFA results have implications for scale construct validity and potential directions for further research or refinement of the scale. Second, we did not do test–retest. Third, the surveyed participants were mostly high educated, urban-based and young (mean age of 35), further testing among current smokers with other demo-social backgrounds or smokers living in other regions or countries is needed. Fourth, volunteer bias may exist due to the recruitment method of advertisements and referrals. Fifth, social desirability bias may exist, similar to the existing smoking cessation literature. Sixth, we did subgroup analyses on gender, and we found some cross factor loading when restricting the data to female smokers. Thus. Further refinements are needed when using this scale among female smokers. Last, this is a cross-sectional design including cognitions in the pre-intentional phase and post-intentional phase. It seems necessary to design longitudinal studies to obtain additional support for its psychometric properties within the populations.

## Conclusion

The present study developed 26-item HAPA-based assessments for smoking cessation among current smokers in China, and the results suggested that these HAPA-based measurements are comprehensive, reliable and valid in the assessment of smokers’ smoking cessation cognitions. We believe that the present full-measurement HAPA scales may be of practical help in assessing the beliefs of current smokers in order to put more effective interventions into action. Moreover, this research extended HAPA to the domain of smoking cessation, which provides opportunities for theory development.

### Supplementary Information

Supplementary Tables.

## Data Availability

The datasets generated during and analysed during the current study are available from the corresponding author on reasonable request.

## References

[1] Teo KK, Rafiq T (2021). Cardiovascular risk factors and prevention: A perspective from developing countries. Can. J. Cardiol..

[2] WHO (2018). WHO Global Report on Trends in Prevalence of Tobacco Smoking 2000–2025.

[3] Zhu D, Zhao G, Wang X (2021). Association of smoking and smoking cessation with overall and cause-specific mortality. Am. J. Prev. Med..

[4] West R (2017). Tobacco smoking: Health impact, prevalence, correlates and interventions. Psychol. Health.

[5] Babb S, Malarcher A, Schauer G, Asman K, Jamal A (2017). Quitting smoking among adults—United States, 2000–2015. MMWR Morb. Mortal. Wkly. Rep..

[6] Beaglehole R, Bates C, Youdan B, Bonita R (2019). Nicotine without smoke: Fighting the tobacco epidemic with harm reduction. Lancet.

[7] Lee SH, Yi YH, Lee YI, Lee HY, Lim KM (2022). Factors associated with long-term smoking relapse in those who succeeded in smoking cessation using regional smoking cessation programs. Medicine.

[8] Nilsen P (2015). Making sense of implementation theories, models and frameworks. Implement. Sci..

[9] Davis R, Campbell R, Hildon Z, Hobbs L, Michie S (2015). Theories of behaviour and behaviour change across the social and behavioural sciences: A scoping review. Health Psychol. Rev..

[10] Lareyre O, Gourlan M, Stoebner-Delbarre A, Cousson-Gélie F (2021). Characteristics and impact of theory of planned behavior interventions on smoking behavior: A systematic review of the literature. Prev. Med..

[11] Lin H, Chen M, Yun Q, Zhang L, Chang C (2021). Tobacco dependence affects determinants related to quitting intention and behaviour. Sci. Rep..

[12] Sabzmakan L, Ghasemi M, Asghari Jafarabadi M, Kamalikhah T, Chaleshgar Kordasiabi M (2018). Factors associated with tobacco use among Iranian adolescents: An application of protection motivation theory. Subst. Use Misuse.

[13] Dai S (2021). Parental knowledge, attitude, and practice on tobacco use, smoking cessation, and children’s environmental tobacco smoke exposure. Front. Public Health.

[14] Ravi K, Indrapriyadharshini K, Madankumar PD (2021). Application of health behavioral models in smoking cessation—A systematic review. Indian J. Public Health.

[15] Schwarzer R (1999). Self-regulatory processes in the adoption and maintenance of health behaviors. J. Health Psychol..

[16] Veilleux JC, Steggerda JC (2023). The dynamics of smoking quit motivation in daily life: Associations with momentary self-regulation and nightly quit intentions. Addict. Behav..

[17] Schwarzer R (2008). Modeling health behavior change: How to predict and modify the adoption and maintenance of health behaviors. Appl. Psychol..

[18] Zhang CQ, Zhang R, Schwarzer R, Hagger MS (2019). A meta-analysis of the Health Action Process Approach. Health Psychol..

[19] Schwarzer R, Lippke S, Luszczynska A (2011). Mechanisms of health behavior change in persons with chronic illness or disability: The Health Action Process Approach (HAPA). Rehabil. Psychol..

[20] Radtke T, Kaklamanou D, Scholz U, Hornung R, Armitage CJ (2014). Are diet-specific compensatory health beliefs predictive of dieting intentions and behaviour?. Appetite.

[21] Zhou S (2021). Physical activity under stress: A perspective of HAPA and individual differences. Int. J. Environ. Res. Public Health.

[22] Araújo MR, Alvarez MJ, Godinho CA, Almeida T, Pereira CR (2020). Self-regulation in oral hygiene behaviours in adults with gingivitis: The mediating role of coping planning and action control. Int. J. Dent. Hyg..

[23] Berli C (2015). Volitional processes and daily smoking: Examining inter- and intraindividual associations around a quit attempt. J. Behav. Med..

[24] Williams RJ, Herzog TA, Simmons VN (2011). Risk perception and motivation to quit smoking: A partial test of the Health Action Process Approach. Addict. Behav..

[25] Scholz U, Nagy G, Göhner W, Luszczynska A, Kliegel M (2009). Changes in self-regulatory cognitions as predictors of changes in smoking and nutrition behaviour. Psychol. Health.

[26] Schwarzer R, Luszczynska A (2008). How to overcome health-compromising behaviors. Eur. Psychol..

[27] Salhi L, Seidel L, Albert A, Lambert F (2021). Fagerström test for nicotine dependence as an indicator in tobacco-related studies in periodontology. J. Periodontol..

[28] Joveini H (2019). The effects of an education program on hookah smoking cessation in university students: An application of the health action process approach (HAPA). J. Subst. Use.

[29] Westmaas JL, Bontemps-Jones J, Bauer JE (2010). Social support in smoking cessation: Reconciling theory and evidence. Nicotine Tob. Res..

[30] Yonek JC, Meacham MC, Shumway M, Tolou-Shams M, Satre DD (2021). Smoking reduction is associated with lower alcohol consumption and depressive symptoms among young adults over one year. Drug Alcohol Depend..

[31] Zvolensky MJ (2020). Anxiety symptoms and smoking among Latinx adult smokers: The importance of sensitivity to internal cues in terms of dependence, barriers for quitting, and quit problems. J. Behav. Med..

[32] Thoits PA (2011). Mechanisms linking social ties and support to physical and mental health. J. Health Soc. Behav..

[33] González P (2017). Measurement properties of the Center for Epidemiologic Studies Depression Scale (CES-D 10): Findings from HCHS/SOL. Psychol. Assess..

[34] Toussaint A (2020). Sensitivity to change and minimal clinically important difference of the 7-item Generalized Anxiety Disorder Questionnaire (GAD-7). J. Affect. Disord..

[35] Alidosti M, Shahnazi H, Heidari Z, Zamani-Alavijeh F (2022). Development and psychometric assessment of cutaneous leishmaniasis prevention behaviors questionnaire in adolescent female students: Application of integration of cultural model and extended parallel process model. PLoS ONE.

[36] Šimkovic M, Träuble B (2019). Robustness of statistical methods when measure is affected by ceiling and/or floor effect. PLoS ONE.

[37] Izquierdo I, Olea J, Abad FJ (2014). Exploratory factor analysis in validation studies: Uses and recommendations. Psicothema.

[38] Konishi T (2015). Principal component analysis for designed experiments. BMC Bioinform..

[39] Rosen RC (1997). The international index of erectile function (IIEF): A multidimensional scale for assessment of erectile dysfunction. Urology.

[40] Kebede Y, Alemayehu G, Abebe L, Sudhakar M, Birhanu Z (2021). Messenger students' engagement scale: Community perspectives on school-based malaria education in Ethiopia. Health Soc. Care Community.

[41] Schreiber JB (2021). Issues and recommendations for exploratory factor analysis and principal component analysis. Res. Soc. Adm. Pharm..

[42] Cao W, Mo PK, Lau JT (2019). Validation of the outcome expectancy scale for HIV serostatus disclosure to female sex partners among men who have sex with men and women living with HIV in China. J. Sex Marital Ther..

[43] Lim SS (2012). A comparative risk assessment of burden of disease and injury attributable to 67 risk factors and risk factor clusters in 21 regions, 1990–2010: A systematic analysis for the Global Burden of Disease Study 2010. Lancet.

[44] Alexandrov LB (2016). Mutational signatures associated with tobacco smoking in human cancer. Science.

[45] Wu JC, Rhee JW, Sallam K (2019). Electronic cigarettes: Where there is smoke there is disease. J. Am. Coll. Cardiol..

[46] Dalton MA, Sargent JD, Beach ML, Bernhardt AM, Stevens M (1999). Positive and negative outcome expectations of smoking: Implications for prevention. Prev. Med..

[47] Reesor L, Vaughan EM, Hernandez DC, Johnston CA (2017). Addressing outcomes expectancies in behavior change. Am. J. Lifestyle Med..

[48] Lee CK, Corte C, Stein KF, Feng JY, Liao LL (2020). Alcohol-related cognitive mechanisms underlying adolescent alcohol use and alcohol problems: Outcome expectancy, self-schema, and self-efficacy. Addict. Behav..

[49] Lin MP, Ko HC, Wu JY (2008). The role of positive/negative outcome expectancy and refusal self-efficacy of Internet use on Internet addiction among college students in Taiwan. Cyberpsychol. Behav..

[50] Carcioppolo N, Orrego Dunleavy V, Myrick JG (2019). A closer look at descriptive norms and indoor tanning: Investigating the intermediary role of positive and negative outcome expectations. Health Commun..

[51] Holahan CJ (2012). Social influences on smoking in middle-aged and older women. Psychol. Addict. Behav..

[52] Sheeran P (2016). The impact of changing attitudes, norms, and self-efficacy on health-related intentions and behavior: A meta-analysis. Health Psychol..

[53] Elwyn G, Frosch DL, Kobrin S (2016). Implementing shared decision-making: Consider all the consequences. Implement. Sci..

[54] Wee ZQC, Dillon D (2022). Increasing physical exercise through action and coping planning. Int. J. Environ. Res. Public Health.

[55] Pribadi ET, Devy SR (2020). Application of the Health Belief Model on the intention to stop smoking behavior among young adult women. J. Public Health Res..

[56] Thrul J, Stemmler M, Bühler A, Kuntsche E (2013). Adolescents' protection motivation and smoking behaviour. Health Educ. Res..

[57] Qian Y, Gui W, Ma F, Dong Q (2021). Exploring features of social support in a Chinese online smoking cessation community: A multidimensional content analysis of user interaction data. Health Inform. J..

[58] Weinberger AH (2017). Depression and cigarette smoking behavior: A critical review of population-based studies. Am. J. Drug Alcohol Abuse.

