# Supplementary material for: Development and psychometric assessment of Health Action Process Approach (HAPA) in terms of smoking cessation among Chinese smokers
Source: Sci Rep. 2024 Feb 19;14:4056. doi: 10.1038/s41598-024-54404-2 (PMC10876652; doi:10.1038/s41598-024-54404-2)
Supplement: Supplementary file 1 — Supplementary Tables. [file 41598_2024_54404_MOESM1_ESM.docx]

**Development and psychometric assessment of Health Action Process Approach (HAPA) in terms of smoking cessation among Chinese smokers**

**Supplementary Tables**

**Supplementary Table 1. Exploratory factor analysis for the Health Action Process Approach among male smokers.**

| **Construct** | | **Factor 1** | **Factor 2** | **Factor 3** | **Factor 4** | **Factor 5** |
| --- | --- | --- | --- | --- | --- | --- |
| **Risk perception in smoking-induced cancer** | | | | | | |
| A1 | What are my chances of getting lung cancer due to smoking? | 0.05 | **0.94** | 0.00 | -0.04 | -0.07 |
| A2 | What are my chances of getting month cancer due to smoking? | 0.00 | **0.96** | -0.01 | 0.00 | -0.01 |
| A3 | What are my chances of getting bladder cancer due to smoking? | -0.09 | **0.90** | 0.03 | 0.03 | 0.12 |
| **Risk perception in smoking-induced systemic disease** | | | | | | |
| B1 | What are my chances of having cardiovascular diseases due to smoking? | 0.06 | **0.95** | -0.01 | -0.01 | -0.05 |
| B2 | What are my chances of having respiratory diseases due to smoking? | 0.06 | **0.93** | -0.01 | -0.05 | -0.08 |
| B3 | What are my chances of having reproductive diseases due to smoking? | -0.02 | **0.89** | 0.01 | 0.03 | 0.09 |
| **Positive outcome expectancy if quit smoking** | | | | | | |
| C1 | My physical condition will be improved | -0.03 | 0.03 | 0.01 | **-0.94** | 0.02 |
| C2 | My smoking-related expenses will be reduced | 0.00 | -0.01 | 0.00 | **-0.96** | 0.00 |
| C3 | My family and friends will be happy | 0.02 | -0.02 | 0.00 | **-0.96** | -0.02 |
| **Negative outcome expectancy if quit smoking** | | | | | | |
| D1 | Stop smoking prevents me from socialization | -0.01 | -0.04 | **0.90** | 0.00 | 0.10 |
| D2 | Stop smoking deprives me of an opportunity for enjoyment | 0.04 | -0.02 | **0.94** | 0.03 | -0.02 |
| D3 | Stop smoking makes me disable to deal with stress | -0.02 | 0.06 | **0.91** | -0.04 | -0.08 |
| **Self-efficacy in quitting smoking** | | | | | | |
| E1 | I can start quitting smoking, even if I feel tense and nervous | **0.97** | 0.00 | 0.03 | 0.01 | -0.05 |
| E2 | I can start quitting smoking, even if I have a strong temptation to smoke | **0.96** | 0.01 | 0.01 | 0.02 | -0.02 |
| E3 | I can start quitting smoking, even if my significant others do not support me to quit smoking | **0.98** | -0.02 | -0.01 | 0.01 | -0.03 |
| **Self-efficacy in maintaining smoking cessation** | | | | | | |
| F1 | I can continue not to smoke, even if I have severe withdrawal symptoms | **1.00** | -0.01 | 0.00 | -0.02 | -0.06 |
| F2 | I can continue not to smoke, even if I feel tense or restless or tired | **0.98** | 0.00 | 0.01 | 0.00 | -0.03 |
| F3 | I can continue not to smoke, even if my friends offer me a cigarette | **0.68** | 0.03 | 0.00 | -0.05 | 0.22 |
| **Self-efficacy in re-initiating smoking cessation after relapse** | | | | | | |
| G1 | I can quit again, even if I have postponed my cessation program several times | **0.71** | 0.07 | -0.02 | -0.03 | 0.22 |
| G2 | I can quit again, even if I am not able to refrain from smoking sometimes | **0.72** | 0.07 | -0.03 | -0.04 | 0.19 |
| G3 | I can quit again, even if I returned to smoking and abandoned the cessation program | **0.70** | 0.05 | -0.03 | -0.06 | 0.22 |
| **Quit smoking planning** | | | | | | |
| H1 | I have a precise plan concerning the time of initiating smoking cessation | 0.04 | 0.02 | 0.00 | -0.01 | **0.92** |
| H2 | I have a precise plan concerning the process of initiating smoking cessation | 0.02 | 0.00 | 0.00 | 0.00 | **0.96** |
| **Coping planning in maintaining smoking cessation** | | | | | | |
| I1 | I have a clear plan on how to avoid smoking places | 0.04 | 0.02 | 0.00 | -0.03 | **0.92** |
| I2 | I have a clear plan on how to cope with temptations to smoke | 0.05 | -0.02 | 0.00 | -0.02 | **0.93** |
| I3 | I have a clear plan on how to overcome the situation which makes me more likely to start smoking again | 0.02 | 0.04 | 0.01 | -0.02 | **0.92** |
| **Cronbach's alpha** | | 0.983 | 0.969 | 0.908 | 0.949 | 0.984 |
| **Initial eigenvalues** | | 14.48 | 3.52 | 2.62 | 1.67 | 0.90 |
| **Cumulative % of variance explained** | | 55.70 | 69.23 | 79.30 | 85.71 | 89.16 |
| Note: Extraction Method: Principal Component Analysis. Rotation Method: Oblimin with Kaiser Normalization. Rotation converged in 7 iterations. | | | | | | |

**Supplementary Table 2. Exploratory factor analysis for the Health Action Process Approach among female smokers.**

| **Construct** | | **Factor 1** | **Factor 2** | **Factor 3** | **Factor 4** | **Factor 5** |
| --- | --- | --- | --- | --- | --- | --- |
| **Risk perception in smoking-induced cancer** | | | | | | |
| A1 | What are my chances of getting lung cancer due to smoking? | 0.00 | -0.02 | **0.93** | 0.05 | 0.01 |
| A2 | What are my chances of getting month cancer due to smoking? | 0.00 | -0.02 | **0.97** | -0.01 | -0.01 |
| A3 | What are my chances of getting bladder cancer due to smoking? | 0.01 | 0.01 | **0.98** | -0.02 | 0.03 |
| **Risk perception in smoking-induced systemic disease** | | | | | | |
| B1 | What are my chances of having cardiovascular diseases due to smoking? | -0.03 | 0.01 | **0.98** | 0.00 | -0.03 |
| B2 | What are my chances of having respiratory diseases due to smoking? | -0.04 | -0.01 | **0.94** | 0.03 | -0.04 |
| B3 | What are my chances of having reproductive diseases due to smoking? | 0.09 | 0.02 | **0.89** | -0.04 | 0.00 |
| **Positive outcome expectancy if quit smoking** | | | | | | |
| C1 | My physical condition will be improved | 0.03 | 0.00 | -0.01 | **0.97** | 0.01 |
| C2 | My smoking-related expenses will be reduced | 0.01 | 0.00 | 0.00 | **0.99** | 0.02 |
| C3 | My family and friends will be happy | -0.03 | 0.01 | 0.01 | **0.98** | -0.01 |
| **Negative outcome expectancy if quit smoking** | | | | | | |
| D1 | Stop smoking prevents me from socialization | 0.01 | **0.97** | 0.01 | -0.01 | -0.02 |
| D2 | Stop smoking deprives me of an opportunity for enjoyment | 0.00 | **0.97** | -0.02 | 0.02 | 0.01 |
| D3 | Stop smoking makes me disable to deal with stress | 0.00 | **0.98** | 0.01 | -0.01 | 0.01 |
| **Self-efficacy in quitting smoking** | | | | | | |
| E1 | I can start quitting smoking, even if I feel tense and nervous | -0.03 | 0.02 | 0.05 | 0.03 | **-0.95** |
| E2 | I can start quitting smoking, even if I have a strong temptation to smoke | -0.02 | -0.01 | 0.03 | 0.07 | **-0.92** |
| E3 | I can start quitting smoking, even if my significant others do not support me to quit smoking | -0.01 | 0.00 | 0.05 | 0.01 | **-0.95** |
| **Self-efficacy in maintaining smoking cessation** | | | | | | |
| F1 | I can continue not to smoke, even if I have severe withdrawal symptoms | 0.03 | 0.01 | 0.04 | 0.01 | **-0.93** |
| F2 | I can continue not to smoke, even if I feel tense or restless or tired | 0.04 | 0.02 | 0.01 | -0.02 | **-0.95** |
| F3 | I can continue not to smoke, even if my friends offer me a cigarette | **0.58** | 0.01 | -0.02 | 0.08 | -0.39 |
| **Self-efficacy in re-initiating smoking cessation after relapse** | | | | | | |
| G1 | I can quit again, even if I have postponed my cessation program several times | **0.55** | -0.01 | 0.03 | 0.00 | -0.45 |
| G2 | I can quit again, even if I am not able to refrain from smoking sometimes | **0.59** | -0.02 | -0.01 | 0.03 | -0.44 |
| G3 | I can quit again, even if I returned to smoking and abandoned the cessation program | **0.58** | -0.02 | 0.00 | 0.02 | -0.42 |
| **Quit smoking planning** | | | | | | |
| H1 | I have a precise plan concerning the time of initiating smoking cessation | **0.95** | -0.01 | 0.02 | 0.03 | 0.04 |
| H2 | I have a precise plan concerning the process of initiating smoking cessation | **0.95** | 0.00 | 0.06 | 0.00 | 0.04 |
| **Coping planning in maintaining smoking cessation** | | | | | | |
| I1 | I have a clear plan on how to avoid smoking places | **0.96** | 0.00 | 0.05 | 0.02 | 0.04 |
| I2 | I have a clear plan on how to cope with temptations to smoke | **0.96** | 0.01 | 0.04 | 0.01 | 0.03 |
| I3 | I have a clear plan on how to overcome the situation which makes me more likely to start smoking again | **0.94** | -0.01 | 0.05 | 0.04 | 0.03 |
| **Cronbach's alpha** | | 0.983 | 0.969 | 0.980 | 0.976 | 0.990 |
| **Initial eigenvalues** | | 15.06 | 3.02 | 2.63 | 2.11 | 1.24 |
| **Cumulative % of variance explained** | | 57.92 | 69.55 | 79.66 | 87.78 | 92.55 |
| Note: Extraction Method: Principal Component Analysis. Rotation Method: Oblimin with Kaiser Normalization. Rotation converged in 9 iterations. | | | | | | |

**Supplementary Table 3. Corrected Item-Total Correlation of the Health Action Process Approach.**

| **Subscale** | **Item number** | **Corrected Item-Total Correlation** |
| --- | --- | --- |
| Risk perception in smoking | A1 | 0.670*** |
|  | A2 | 0.671*** |
|  | A3 | 0.661*** |
|  | B1 | 0.688*** |
|  | B2 | 0.674*** |
|  | B3 | 0.673*** |
| Positive outcome expectancy if quit smoking | C1 | 0.553*** |
|  | C2 | 0.549*** |
|  | C3 | 0.540*** |
| Negative outcome expectancy if quit smoking | D1 | 0.183*** |
|  | D2 | 0.126*** |
|  | D3 | 0.124*** |
| Self-efficacy in quitting, maintaining and re-initiating smoking cessation | E1 | 0.813*** |
|  | E2 | 0.831*** |
|  | E3 | 0.817*** |
|  | F1 | 0.838*** |
|  | F2 | 0.829*** |
|  | F3 | 0.833*** |
|  | G1 | 0.865*** |
|  | G2 | 0.865*** |
|  | G3 | 0.861*** |
| Planning in quit smoking and maintaining smoking cessation | H1 | 0.813*** |
|  | H2 | 0.813*** |
|  | I1 | 0.822*** |
|  | I2 | 0.812*** |
|  | I3 | 0.822*** |

Note: ****p*<0.001.
